# GalNAc-T14 promotes metastasis through Wnt dependent *HOXB9* expression in lung adenocarcinoma

**DOI:** 10.18632/oncotarget.6019

**Published:** 2015-10-26

**Authors:** Ok-Seon Kwon, Ensel Oh, Jeong-Rak Park, Ji-Seon Lee, Gab-Yong Bae, Jae-Hyung Koo, Hyongbum Kim, Yoon-La Choi, Young Soo Choi, Jhingook Kim, Hyuk-Jin Cha

**Affiliations:** ^1^ College of Natural Sciences, Department of Life Sciences, Sogang University, Seoul, Republic of Korea; ^2^ Laboratory of Cancer Genomics and Molecular Pathology, Samsung Biomedical Research Institute, Samsung Medical Center, Seoul, Republic of Korea; ^3^ Department of Brain and Cognitive Sciences, DGIST Daegyu, Republic of Korea; ^4^ Department of Pharmacology, Yonsei University College of Medicine, Seoul; ^5^ Department of Pathology, Samsung Medical Center, Sungkyunkwan University, School of Medicine, Seoul, Republic of Korea; ^6^ Department of Thoracic Surgery, Samsung Medical Center, Sungkyunkwan University, School of Medicine, Seoul, Republic of Korea

**Keywords:** metastasis, GalNAc-T14, WNT/TCF pathway, *HOXB9*, invasion

## Abstract

While metastasis, the main cause of lung cancer-related death, has been extensively studied, the underlying molecular mechanism remains unclear. A previous clinicogenomic study revealed that expression of N-acetylgalactosaminyltransferase (GalNAc-T14), is highly inversely correlated with recurrence-free survival in those with non-small cell lung cancer (NSCLC). However, the underlying molecular mechanism(s) has not been determined. Here, we showed that GalNAc-T14 expression was positively associated with the invasive phenotype. Microarray and biochemical analyses revealed that *HOXB9*, the expression of which was increased in a GalNAc-T14-dependent manner, played an important role in metastasis. GalNAc-T14 increased the sensitivity of the WNT response and increased the stability of the β-catenin protein, leading to induced expression of *HOXB9* and acquisition of an invasive phenotype. Pharmacological inhibition of β-catenin in GalNAc-T14-expressing cancer cells suppressed *HOXB9* expression and invasion. A meta-analysis of clinical genomics data revealed that expression of GalNAc-T14 or *HOXB9* was strongly correlated with reduced recurrence-free survival and increased hazard risk, suggesting that targeting β-catenin within the GalNAc-T14/WNT/*HOXB9* axis may be a novel therapeutic approach to inhibit metastasis in NSCLC.

## INTRODUCTION

Lung cancer is not only the most commonly diagnosed cancer but also the leading cause of cancer-associated death, due to its poor prognosis and active metastasis to other organs after diagnosis [1]. Suppression of metastasis are therefore being investigated as future therapeutic strategies [2]. However, the molecular mechanisms of lung cancer metastasis are largely unknown, and therefore, putative molecular targets remain to be identified.

Wnt signaling, one of the most well-characterized signaling pathways in cancer metastasis [3], is activated by Wnt ligand binding to the Frizzled receptor, which stabilizes β-catenin. β-catenin, in turn, associates with the T cell factor (TCF)/lymphoid enhancing factor (LEF) transcription factors to induce the expression of a number of genes involved in cancer cell proliferation, survival [4], and metastasis [5]. For example, *HOXB9*, a class 1 homeobox gene associated with tumorigenesis, metastasis, and poor prognosis [6, 7], is upregulated by Wnt signaling and promotes lung cancer metastasis [8]. Therefore, targeting β-catenin to inhibit cancer growth and metastasis has been extensively studied, and several small molecule inhibitors of β-catenin are in clinical trials [9, 10].

The N-acetyl-galactosaminyl-transferases (GalNAc-Ts or GalNTs) are responsible for synthesizing the Thomsen-nouvelle antigen (Tn), one of cancer-associated O-glycans [11, 12], whose expression is positively associated with many types of cancer and correlates with metastasis and even poor survival [13–16]. Thus, GalNAc-Ts such as GalNAc-T2 [17–19], T13 [20], and T14 [21] were shown to induce metastatic potential. Of interest, GalNAc-T isoenzymes produce unique O-glycoproteomes of signaling molecules such as a death receptor [22, 23], implying that GalNAc-Ts expression in cancer cells may affect the cellular response to external stimuli such as growth factors, cytokines to determine cancer-associated phenotypes.

Previously, we reported that expression of GalNAc-T14 (or GALNT14) was highly associated with poor recurrence-free survival in a clinicogenomics study with 138 non-small cell lung cancer (NSCLC) patients [24]. However, the molecular mechanism for the significant difference in poor recurrence-free survival remained undetermined. Here, we show that GalNAc-T14 increases β-catenin protein stability and the subsequent Wnt target gene response, particularly the induction of *HOXB9*, the expression of which is critical for the invasive properties of lung adenocarcinoma. In addition, pharmacological inhibition of β-catenin significantly suppressed invasive features, suggesting that treatment of GalNAc-T14-expressing lung cancers with a β-catenin inhibitor would be a valid therapeutic approach to reduce their metastatic potential.

## RESULTS

### Loss of GalNAc-T14 results in retarded migration and invasiveness

**Figure 1:**
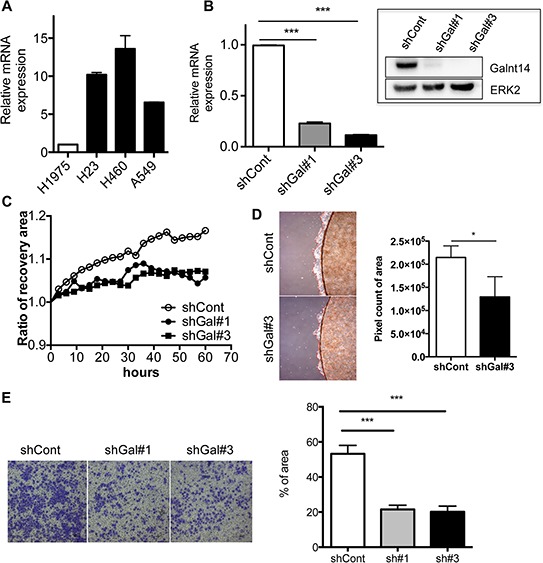
Loss of GalNAc-T14 results in retarded migration and invasiveness **A.** GalNAc-T14 mRNA expression in NSCLC cell lines H1975, H23, H460 and A549 was determined via real-time PCR (*n* = 2). **B.** Level of GalNAc-T14 mRNA and protein (inserted panel) in shGal#1 and shGal#3 cells, ERK2 a loading control **C.** Time dependent cell migration rate in shCont, shGal#1 and shGal#3 after serum starvation was shown in graph. Recovery ratio was measured every 6 hrs. **D.** Cell migration rate from the coverslip to the empty area, determined by measuring 5 random areas (red dotted line, right panel) after 4 days. Total pixel count of the area of cell migration (red dotted line) was presented as a bar graph (left panel). **E.** Representative image of cells, invaded through trans-well membrane (left panel) was shown. Quantification of invaded cells was shown in a bar graph (right panel) (*n* = 5).

To investigate the role of GalNAc-T14 in lung cancer metastasis, the GalNAc-T14 expression levels in NSCLC cell lines was first compared. As shown previously [23], H460 expressed a higher level of GalNAc-T14 compared to H1975 (Fig. 1A). Thus, GalNAc-T14 knockdown cells were generated by the stable expression of GalNAc-T14 shRNA (shGalNAc-T14; shGal) in H460 cells. Among the five shRNAs for GalNAc-T14, two independent shRNAs (#1 and 3) suppressed GalNAc-T14 efficiently (Fig. S1A), and stable expression of each shRNA produced two clones of GalNAc-T14 knockdown H460 cells (shGal#1 and shGal#3; shGal-H460) (Fig. 1B). As expression of GalNAc-T14 is most significantly high in patients with lung cancer recurrence [24], we hypothesized that GalNAc-T14 expression may promote lung cancer metastasis. As predicted, the migration capacity of both the shGal#1 and shGal#3-H460 cell lines, determined by measuring the recovery area in a scratch assay, was markedly decreased compared to the control (Fig. 1C), while the cell proliferation rate remained unaltered in the shGal-H460 lines (Fig. S1B). In addition, cell migration of confluent cells grown on a coverslip toward the empty area of the cell plate was also significantly weakened in the shGal#3 cell line (Fig. 1D). As not only the movement on 2D surface but also the infiltration through 3D matrix is critical capacity of tumor cells to promote metastasis, the invasive characteristics of the cell lines were compared in a two-chamber invasion assay. As shown in Fig. 1E, lack of GalNAc-T14 also reduced the invasive properties of H460 cells. Taken together, these data imply that GalNAc-T14 expression increases the metastatic potential of lung cancer cells.

### GalNAc-T14 controls Wnt responsiveness

Next, to examine the molecular mechanism through which GalNAc-T14 controls metastatic potential, a microarray analysis was performed to compare gene expression in the two shGal-H460 cell lines compared to the parental control cells (Fig. S2A). The commonly altered gene set in the two shGal-H460 cell lines was analyzed by both gene ontology and gene card analysis. Of interest, 29.1% of altered genes in both shGal#1 and #3 are related to metastasis (i.e., invasion and migration), supporting the results in Fig. 1 that GalNAc-T14 expression is linked to metastatic potential (Fig. S2).

**Figure 2:**
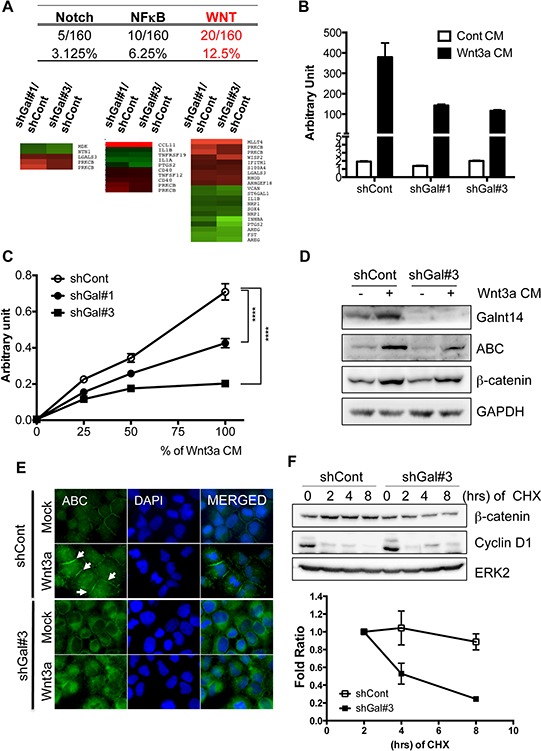
GalNAc-T14 controls Wnt responsiveness **A.** Percentage (top panel) and heat map (bottom pane) of genes in each signaling pathway (NF-kB, Notch and Wnt), commonly altered in shGal#1 and shGal#3 was shown. **B.** Reporter activity by TOPflash assay with or without Wnt3a CM (50%) was presented as a bar graph. **C.** Reporter activity by TOPflash assay after indicative dose of Wnt3a CM (%) was shown. **D.** Active or total β-catenin protein level after 50% of Wnt3a CM treatment, was determined by immunoblotting analysis. ERK2 for a loading control, **E.** Cells were stained with ABC antibody (green) and DAPI (blue) after Wnt3a CM treatment for 24 hours, White arrows indicate membrane-associated β-catenin. **F.** Time dependent protein levels of β-catenin and cyclin D1 after treatment of cycloheximide (CHX: 40 μg/ml) were determined (top panel). ERK2 was used for an equal loading control. Relative band intensity of β-catenin was graphically presented (bottom panel) (*n* = 2).

To identify a signaling pathway governing GalNAc-T14-dependent metastatic potential from among a number of signaling pathways underlying metastasis [25, 26], the altered gene expression profile was carefully reanalyzed. Of note, the two main sets of genes altered by the lack of GalNAc-T14 are those involved in metastasis (29.1%) and stemness (20.6%) (Fig. S2B). Therefore, we focused on the NF-kB [27], Notch [28], and Wnt [29] signaling pathways, which are implicated in cancer stemness as well as metastasis. Through analysis of the altered gene set in the microarray data (Fig. 2A) and following assessment of reporter activity (Fig. 2B and S3A/SB), we concluded that Wnt activity was most significantly reduced by GalNAc-T14 knock-down. As shown in Fig. 2B, Wnt reporter activity in both shGal#1 and shGal#3 was markedly reduced upon Wnt3a supplementation [Wnt3a conditioned medium (Wnt3a CM)] compared to controls. Similarly, dose-dependent Wnt reporter activity in the absence of GalNAc-T14 was also notably decreased compared to the control (Fig. 2C). It is noteworthy that Wnt responsiveness, shown in Fig. 2C, appeared to be adversely correlated with the level of GalNAc-T14 shown in Fig. 1B (i.e., higher knockdown efficiency in shGal#3 than in shGal#1), suggesting that GalNAc-T14 expression may be important for increased Wnt responsiveness. As Wnt responsiveness results from accumulation of unphosphorylated (active) β-catenin (ABC), which is resistant to protein degradation by the Adenomatous polyposis coli (APC) destruction complex, the level of unphosphorylated β-catenin was determined using an ABC antibody [30]. The increased level of the ABC and nuclear level of β-catenin by Wnt3a supplement in shGal-H460 cells was markedly lower than that of control (Fig. 2D and S3C). Of note, GalNAc-T14 was dominantly located in Golgi complex stained with GM130 unlike ABC in the plasma membrane (Fig. S3D). In support of this result, unphosphorylated β-catenin, recruited to the plasma membrane upon Wnt3a treatment, contributing to Wnt downstream gene response [31], was clearly reduced by GalNAc-T14 knockdown (Fig. 2E, white arrows). As the APC destruction complex recognizes phosphorylated β-catenin for degradation, the protein stability of β-catenin in shGal#3 cells was significantly reduced, whereas cyclin D1 protein stability appeared to be equivalent regardless of GalNAc-T14 expression (Fig. 2F). These data suggest that weakened Wnt responsiveness induced by GalNAc-T14 knockdown would result in lower expression of a metastasis mediator(s) in our model system.

### HOXB9 expression is regulated by GalNAc-T14 through the Wnt pathway

**Figure 3:**
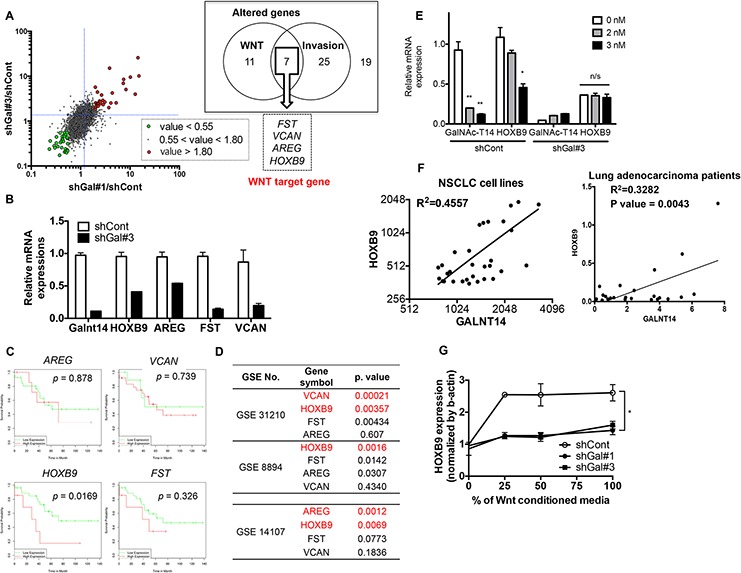
HOXB9 expression is regulated by GalNAc-T14 through the Wnt pathway **A.** Scatter plot for global mRNA expression of shGal#1/shCont and shGal#3/shCont with indicative cut-off range was shown (left panel). List of genes commonly altered in ‘Wnt’ (11 genes) and ‘Invasion’ (25 genes) out of total altered genes (19 genes, cut off range = 1.8) was shown in a box (right panel). **B.** mRNA expression of indicated genes was determined by real time PCR analysis. **C.** KM-plot for indicated 4 genes in lung cancer patients was shown with each *p* value. **D.** *p* value of each indicative gene from GEO database was listed. **E.** mRNA level of GalNAc-T14 and HOXB9 after different dose of siRNA for GalNAc-T14 treatment was determined by real-time PCR analysis (n/s: not significant). **F.** Relative expression level of GalNAc-T14 and HOXB9 mRNA expression in NSCLC cell lines (upper panel) and 23 lung cancer patient samples from TCGA database (lower panel) was shown as a scatter plot with a linear regression graph. **G.** mRNA level of HOXB9 after indicative dose of WNT3a CM treatment, were analyzed by real time PCR analysis.

To identify a putative metastasis mediator, we searched for commonly altered genes in shGal-H460 cells that are not only downstream of the Wnt/TCF pathway but are also associated with metastasis. Among 62 genes, those whose expression was significantly altered in both shGal#1 and #3 cells (cutoff range: greater than 1.8 and less than 0.55), and that are downstream of Wnt and involved in metastasis (including invasion and migration), as defined by either gene ontology, gene card analysis, or literature search, were further selected. Only four genes, follistatin (*FST*), versican (*VCAN*), amphiregulin (*AREG*), and homeobox B9 (*HOXB9*), were found to satisfy these criteria (Figs 3A and S4A). The decreased levels of these four genes in shGal#3 cells were further validated by real-time PCR analysis (Fig. 3B). Amphiregulin (AREG), a well-characterized prognostic and metastatic biomarker of various types of cancers [32, 33], has been extensively studied in malignancy and metastasis [34], validating our approach to identify genes putatively involved in metastasis. To further determine a key target governing GalNAc-T14-dependent metastatic potential, multiple lung cancer clinicogenomics databases for patient overall survival (GSE36471) (Fig. 3C) and cancer stage and recurrence (GSE31210 and GSE8894) (Fig. 3D) were examined. As shown in Fig. 3C, of the four gene candidates, high *HOXB9* expression was found to be the most significantly correlated with reduced patient survival in lung adenocarcinoma, according to at least one publically available clinicogenomics database (GSE36471, http://www.bioprofiling.de/GEO/DRUGSURV/index.html) [35]. Interestingly, in two independent clinicogenomics studies (GSE31210 and GSE8894), *HOXB9* expression was most significantly altered in patients with high-grade tumors (GSE31210) and in recurrent lung cancer (GSE8894), respectively. Moreover, *HOXB9* was also found to be the most significantly increased gene in a microarray database of PC9-BrM3 cells (GSE14107), which are derived from the PC9 lung adenocarcinoma cell line and form brain metastases with close to 100% efficiency (Figs 3C and S4B). Of note, GalNAc-T14 expression was also highly expressed in the same database (Fig. S4B). In a previous study, a total of 138 NSCLC tumor samples were analyzed by microarray, and GalNAc-T14 was the most significant gene out of 20 genes that were highly expressed in lung cancer patients with a high risk of recurrence [24]. Thus, an appealing hypothesis is that *HOXB9* expression may be caused by increased Wnt activity due to high GalNAc-T14 expression in lung cancer, and is responsible for the increased metastatic potential. To test this hypothesis, the dependency of *HOXB9* expression on GalNAc-T14 was first confirmed by siRNA, to rule out any possible non-specific response in shGal-H460 cells. As shown in Fig. 3E, knockdown of GalNAc-T14 by siRNA significantly lowered *HOXB9* expression in control H460 cells but not in shGal#3 cells. Considering the positive correlation between GalNAc-T14 and *HOXB9* in a cell model (Figs 3E and S4B), the potential positive correlation between GalNAc-T14 and *HOXB9* expression was examined in other lung cancer cell models and in cancer tissue. To this end, we took advantage of multiple open access databases (http://www.nextbio.com and http://cancergenome.nih.gov) for cancer cell line and cancer tissue analysis, respectively. Intriguingly, a close positive correlation between *HOXB9* and GalNAc-T14 was observed both in 31 NSCLC cell lines (top panel) and in 23 lung adenocarcinoma patients (bottom panel) (Fig. 3F), implying that GalNAc-T14 expression is closely associated with *HOXB9* expression in lung cancer. More importantly, GalNAc-T14 was evidently responsible for Wnt responsiveness, resulting in *HOXB9* induction upon Wnt supplementation (Fig. 3G).

### HOXB9 expression is responsible for the invasive properties conferred by GalNAc-T14

**Figure 4:**
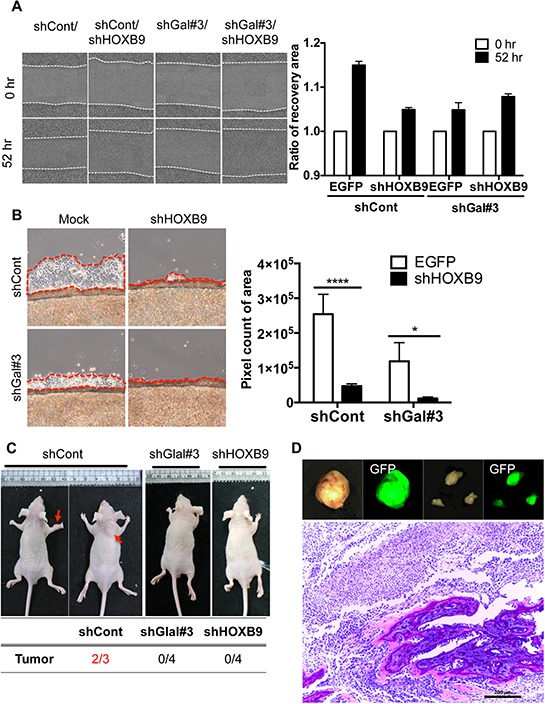
HOXB9 expression is responsible for the invasive properties conferred by GalNAc-T14 **A.** Representative images of recovery were shown after 45 hrs (left panel). Relative recovery area at each condition was presented as a graph (right panel, ns: not significant). **B.** Cell migration rate from the coverslip to the empty area, determined by measuring 5 random areas (red dotted line, right panel) after 4 days. Total pixel count of the area of cell migration (red dotted line) was presented as a bar graph (left panel). **C.** Representative images of mice 6 weeks after tail-vein injection of H460 shCont, shGal#3 and shHOXB9 cell (2 × 10^6^) were shown (top panel, red arrows indicate tumor mass). Summary of tumor formation in each condition was shown in a table (bottom panel) **D.** Tumor tissues, emitting green fluorescence, isolated from mice (GFP pre-labeled shCont H460 cells), were shown (top panel). H&E staining of tumor showing bone metastasis (bottom panel)

As *HOXB9* was the most promising Wnt downstream target to account for the induction of metastatic potential by GalNAc-T14, *HOXB9* knockdown cells were next generated (Fig. S5A), and migration capability with or without GalNAc-T14 expression was monitored. Cell migration (Fig. 4A) but not proliferation capability (Fig. S5B) was significantly suppressed by *HOXB9* knockdown in control H460 cells but not in shGal#3 cells, where *HOXB9* expression was already reduced (Fig. 3B). Similar results were achieved with independent knockdown of *HOXB9* with siRNA (Fig. S5C). In addition, cell mobility from the confluent cell front to the empty area was noticeably decreased by downregulation of *HOXB9*, and was completely inhibited by dual knockdown of *HOXB9* and GalNAc-T14 (Fig. 4B). Not only migration capacity but also invasive property was significantly impaired by *HOXB9* knockdown (Fig. S5D). To finally confirm the effect of GalNAc-T14 and *HOXB9* expression in metastasis *in vivo*, established cell lines, which were further labeled with EGFP for tracking purposes, were injected *i.v*. into mice. Whereas tumor formation was observed at the dorsal area and forelimbs of the control group (two out of three mice), none of the mice that received either the shGal#3 or the sh*HOXB9* cell line developed tumors (Fig. 4C). All tumor tissues, isolated from the shCont group showed clear green fluorescence (Fig. 4D, top panel). In special, tumor isolated from the forelimbs showed clear bone metastasis (Fig. 4D, bottom panel). Collectively, these results indicate that *HOXB9* and GalNAc-T14 expression are strongly correlated with metastatic tumor formation.

### Targeting β-catenin for suppressing metastasis through repression of HOXB9

**Figure 5:**
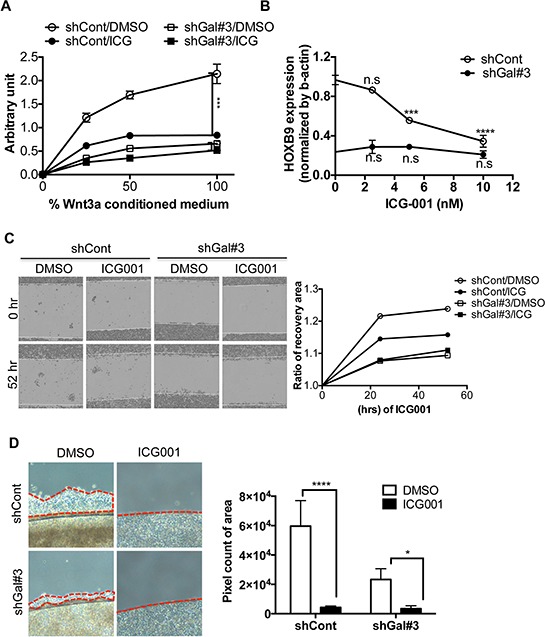
Targeting β-catenin for suppressing metastasis through repression of HOXB9 **A.** Reporter activity by TOPflash assay with or without 10nM of ICG-001 (ICG) treatment after indicative dose of Wnt3a CM (%) was shown. **B.** HOXB9 mRNA level of HOXB9 with indicative dose of ICG-001 (ICG) treatment, was examined by RT-PCR (n.s: not significant). **C.** Representative images of recovery were shown after 45 hrs (left panel). Relative recovery area at each condition was presented as a graph (right panel). **D.** Cell migration rate from the coverslip to the empty area with or without 10nM of ICG-001, determined by measuring 5 random areas (red dotted line, right panel) after 4 days. Total pixel count of the area of cell migration (red dotted line) was presented as a bar graph (left panel).

*HOXB9*, a putative downstream target of Wnt/TCF signaling that was dependent on GalNAc-T14 expression, contributed to metastatic tumor formation (Figs 3 and 4). These results indicated that using a pharmacological inhibitor to target β-catenin, whose protein stability was clearly increased by GalNAc-T14, would be a valid approach to inhibit *HOXB9*-dependent metastasis. To this end, ICG-001, a pharmacological inhibitor of β-catenin, an analog which is currently in clinical trials [9], was used in the lung cancer cell model. Suppression of Wnt reporter activity under Wnt supplementation was evident in the controls, but not in shGal#3 cells (Fig. 5A). Consequently, *HOXB9* expression was also suppressed in an ICG-001 dose-dependent manner in the control cells, but *HOXB9* remained low in shGal#3 cells (Fig. 5B). The migration rate, as determined by wound healing array, was significantly repressed by ICG-001 treatment (Fig. 5C). Similarly, cell migration from confluent cells grown on a coverslip was remarkably reduced by ICG-001 treatment of control cells and reduced to a lesser degree in shGal#3 cells (Fig. 5D). These data strongly suggest that targeting β-catenin in lung cancer with high expression of GalNAc-T14, which sensitizes lung cancer cells to Wnt, leading to *HOXB9* expression, would be a valid approach to suppress *HOXB9*-dependent metastatic potential.

### Prognostic significance of the expression of GalNAc-T14 and HOXB9 in lung cancer

**Figure 6:**
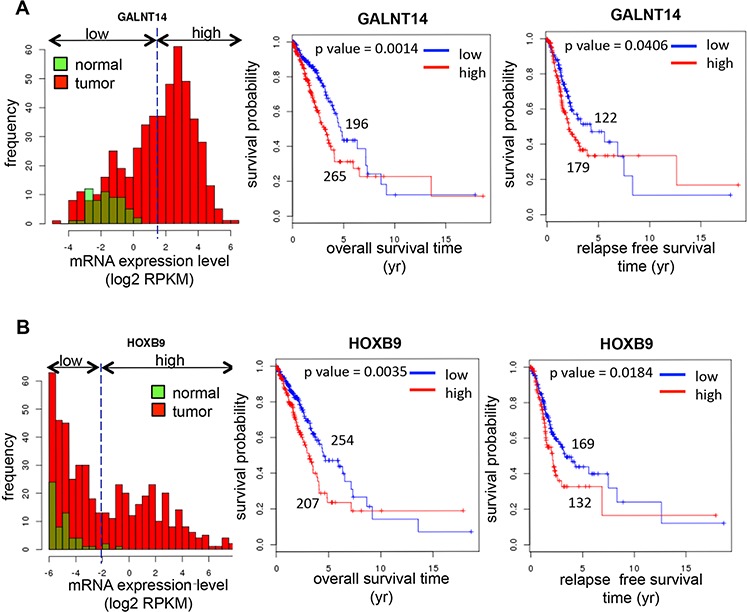
Prognostic significance of expression of GalNAc-T14 and HOXB9 in lung cancer Histogram of the expression levels (left panel), KM overall survival curves (middle panel), and KM relapse-free survival curves (right panel) of GalNAc-T14 **A.** and HOXB9 **B.** from TCGA database analysis was shown respectively. Green or red box indicates normal or tumor respectively.

To investigate the prognostic significance of GalNAc-T14 and *HOXB9*, a survival analysis was performed with large clinical data sets, The Cancer Genome Atlas (TCGA). TCGA data was generated with about 500 lung adenocarcinoma samples using RNA-Seq. In the data set, a considerable number of the cancer samples showed highly increased expression of GalNAc-T14 or *HOXB9* compared to the normal samples, but there were no differences between normal and tumor samples in the expression of the other three candidate genes, *FST*, *VCAN*, and *AREG* (Fig. S6). For survival analysis, the samples were divided into two groups, low and high, according to the expression of GalNAc-T14 and *HOXB9* in normal samples (Figs 6A and 6B). By Kaplan–Meier analysis, the group expressing high levels of GalNAc-T14 (Fig. 6A) or *HOXB9* (Fig. 6B) showed significantly worse outcomes for both overall and relapse-free survival than the low-expressing groups. Moreover, multivariate Cox regression analysis showed that the expression level of GalNAc-T14 and *HOXB9* retained their significance as independent prognostic factors for both overall survival (GalNAc-T14: hazard ratio = 1.731, *p* = 0.003; *HOXB9*: hazard ratio = 1.532, *p* = 0.016) and recurrence (GalNAc-T14: hazard ratio = 1.487, *p* = 0.033; *HOXB9*: hazard ratio = 1.381, *p* = 0.089) (Table A and B for GalNAc-T14 and C and D for *HOXB9*). Taken together, expression level of GalNAc-T14 and *HOXB9* are each associated with the risk of poor overall survival and high recurrence in lung adenocarcinoma patients.

## DISCUSSION

Pharmacological approaches targeting Wnt or β-catenin signaling are being extensively studied for the inhibition of tumor growth and metastasis [36–38]. A few small molecules are currently in clinical trials [9]. For successful targeted therapy of lung cancer with a Wnt/β-catenin inhibitor, a particular lung tumor, which is influenced by Wnt/β-catenin needs to be identified. However, due to the lack of a clear marker for aberrance [e.g., a high β-catenin expression level is paradoxically associated with improved clinical outcome [39, 40]], as well as the diversity of main effectors in Wnt/β-catenin signaling, it will be critical to properly diagnose Wnt/β-catenin dependency for optimal therapeutic selection, as suggested previously [38, 41]

Herein, we demonstrated that GalNAc-T14, the expression of which has been previously correlated with lung cancer recurrence, promoted metastatic potential through Wnt/β-catenin-dependent *HOXB9* expression. Therefore, pharmacological inhibition of β-catenin, in order to block transcriptional factor complex formation, significantly attenuated the metastatic properties of GalNAc-T14-expressing cells (Fig. 5). Given that GalNAc-T14 expression in lung cancer is readily detectable by immunohistochemistry (IHC) [42], GalNAc-T14-positive cells (i.e., the possible metastatic population) would be a valid target for anti-Wnt/β-catenin therapy in order to suppress possible metastasis.

Previously, GalNAc-T14 was shown to promote apoptosis induced by a tumor necrosis factor (TNF)-related apoptosis-inducing ligand (TRAIL) by increasing GalNAc modification of the death receptor (DR), which leads to an increase in death complex formation [23]. As the addition of an N-acetylgalactosamine (GalNAc) moiety onto a signaling molecule such as DR4 by the enzymatic activity of GalNAc-T14 is responsible for altering cellular signaling toward apoptosis [23], it will be important to identify the molecular target of GalNAc, which results in increased Wnt/β-catenin activity and may account for its ability to increase metastatic potential. We also examined the GalNAc status of a number of positive mediators of Wnt/β-catenin signaling. Given that increased GalNAc-T14-dependent Wnt responsiveness was apparent under conditions of Wnt3a supplementation (Figs 2B and 2C), we are currently investigating the possibility that GalNAc status of Wnt receptor/co-receptor by GalNAc-T14 may be responsible for altered Wnt responsiveness, using lectin co-immunoprecipitation experiment, which was used for detecting the alteration of O-glycans of epithelial growth factor receptor by GalNAc-T2 [17] (data not shown). Currently, the identification of a putative GalNAc-T14 target molecule(s) that links GalNAc-T14 to the Wnt/β-catenin/*HOXB9* axis is under investigation.

We also assessed the prognostic significance of GalNAc-T14 and *HOXB9* in patients with lung adenocarcinoma. In a large data set (461 cases for the TCGA), GalNAc-T14 and *HOXB9* expression was apparently associated with an increased risk of poor overall survival and high recurrence in lung adenocarcinoma patients, indicating a possible clinical relevance for their involvement in metastasis.

Taken together, we showed evidence that GalNAc-T14 expression is responsible for Wnt/β-catenin-dependent *HOXB9* expression, which was critical for increased metastatic potential in lung adenocarcinoma. Thus, targeting β-catenin with a small molecule in GalNAc-T14- and *HOXB9*-expressing lung cancers may be an effective therapy to suppress metastatic properties.

## MATERIALS AND METHODS

### Reagents and antibodies

The primary antibody against GalNAcT-14 (16939–1-AP) was acquired from proteintech. The primary antibody against ERK2 (Sc-154), PARP (Sc-7150) and b-actin (Sc-47778) were obtained from Santa Cruz Biotechnology Inc. The primary antibody against total form of b-catenin (Cat.610153) and Active β-catenin (ABC) (Cat.05–665) was purchased from BD Biosciences pharmingen and millipore respectively. ICG-001 (S2662, WNT inhibitor) was purchased from Selleckchem.

### Gene expression profiling

Total RNA was extracted with Trizol (Invitrogen) and the synthesis of target cRNA probes and hybridization were performed using Agilent's Low RNA Input Linear Amplification kit (Agilent Technology, USA) according to the manufacturer's instructions. The fragmented cRNA directly pipetted onto assembled Agilent's Human Oligo Microarray (44K). The arrays hybridized as the manufacturer's protocol. The hybridized images were scanned using Agilent's DNA microarray scanner and quantified with Feature Extraction Software (Agilent Technology, Palo Alto, CA).

### Migration assay

For wound healing assay, after cells were packed in the 6well plate, media was changed without FBS for 24 hrs. A scratch was made on a uniform layer of cells using sterile micropipette tip. Cell migration was monitored daily using microscope and live image were taken by using Incucyte cell live image system (Essen instruments, WelWyn Garden City, UK). For migration assay, coverslip on which cells were plated compactly was transfer to empty 6well plate as previously described [43]. After 4 days cells, which migrated off coverslip were photographed. The pixel count of area of cell migration was obtained with ImageJ software (http://imagej.nih.gov/ij/).

### Survival analysis

Survival analyses were performed with TCGA (The Cancer Genome Atlas, http://cancergenome.nih.gov/) database and on-line survival analysis (Kaplan-Meier plotter and DRUGSV [44]). TCGA is the RNA-Seq V2 data of lung adenocarcinoma. For TCGA data, the number of reads mapping to genes (raw_counts) was normalized using *edgeR* package with statistical software R (http://www.r-project.org/), then the normalized read counts were converted to log2 RPKM (Reads Per Kilobase of transcript per Million mapped reads) values [45, 46]. Kaplan-Meier and Cox regression analyses were performed using *survival* package.

### Animal model for metastasis

shCont, shGal#3 and shHOXB9 cells (2 × 10^6^) were injected into lateral tail vein of male BALB/C nude mice. 4 weeks after injection tumor mass were harvested, fixed with 4% paraformaldehyde for 1 week, embedded in paraffin and stained with hematoxylin and eosin (H&E).

### Statistical analysis

Graphical data were presented as mean ± S.D. Statistical significance among three groups and between groups were determined using one- or two-way analysis of variance (ANOVA) following Bonferroni multiple comparison pos*t*-test and Student's *t*-test, respectively. Significance was assumed for *p* < 0.05 (*), *p* < 0.01 (**), *p* < 0.001 (***).

## SUPPLEMENTARY DATA


