# Supplementary material for: GalNAc-T14 promotes metastasis through Wnt dependent HOXB9 expression in lung adenocarcinoma
Source: Oncotarget. 2015 Oct 26;6(39):41916–28. doi: 10.18632/oncotarget.6019 (PMC4747198; doi:10.18632/oncotarget.6019)
Supplement: Supplementary file 1 [file oncotarget-06-41916-s001.pdf]

SUPPLEMENTARY DATA

Cell culture

H460 cells were viral transfected with pLKO.1 control or shGalNAc-T14 vectors and subsequently selected with puromycin (2 ug/ml) to generate stable cell line and were grown in RPMI 1640 1X supplemented with 10% (v/v) fetal bovine serum (FBS) with 0.1% gentamycin.

Plasmid and siRNA transfection

shRNA plasmid targeting GalNAc-T14 and non-targeting shRNA control plasmid were purchased from Sigma-Aldrich. siRNA targeting GalNAc-T14 and *HOXB9* non targeting siRNA were purchased from Bioneer. Cells were transfected with 20 nM siRNA using either Lipopteatmine 2000 (Invitrogen) or Dharmafect (Thermo Scientific, Waltham, MA).

Trans-well invasion assay

Trans-well (6.5mm) with 8 µm pore polycarbonate membrane insert (Corning, NY, USA) were embedded with 120 µg matrigel (BD Biosciences, San Jose, CA, USA) and 100 ug gelatin (sigma-aldrich) coats bottom of membrane. Cells were added into the Matrigel-embedded insert with serum free RPMI media, and the inserts were placed into the bottom chambers containing 10% FBS media. Cells were incubated for 24–48 hrs. The images were taken with light microscope.

Immunoblotting

Cells were lysed with TLB buffer (20 mM Tris-HCl (pH7.4), 137 mM NaCl, 2 mM EDTA, 1% triton X-100, and 10% glycerol) supplemented with 10 uM sodium vanadate and 1 mM protease inhibitor cocktail (Roche, Basel, Switzerland) and subjected to SDS-PAGE followed by immunoblotting using primary antibodies, and horseradish peroxidase (HRP)-conjugated secondary antibodies (Jackson ImmunoResearch Laboratories, West Grove, PA).

Dual iuciferase assay

Cells were transfected with TOP-Flash and pRL plasmid with Lipopetamine 2000. For 24 hrs incubation with WNT3a conditioned media, reporter assays were performed according to the manufacturer's instructions (Promega). Promoter activity was calculated as the ratio of specific TOP-Flash over pRL.

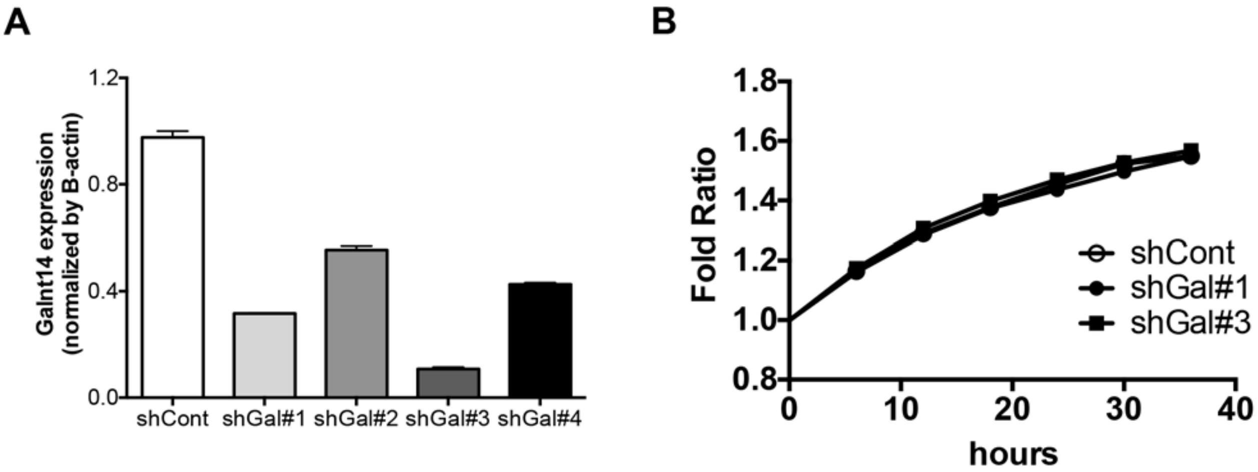

Supplementary Figure S1: A. mRNA level of GalNAc-T14 after knockdown with each different shRNA, was shown in a bar graph. **B.** Cell proliferation rate of shCont, shGal#1 and shGal#3 was shown.

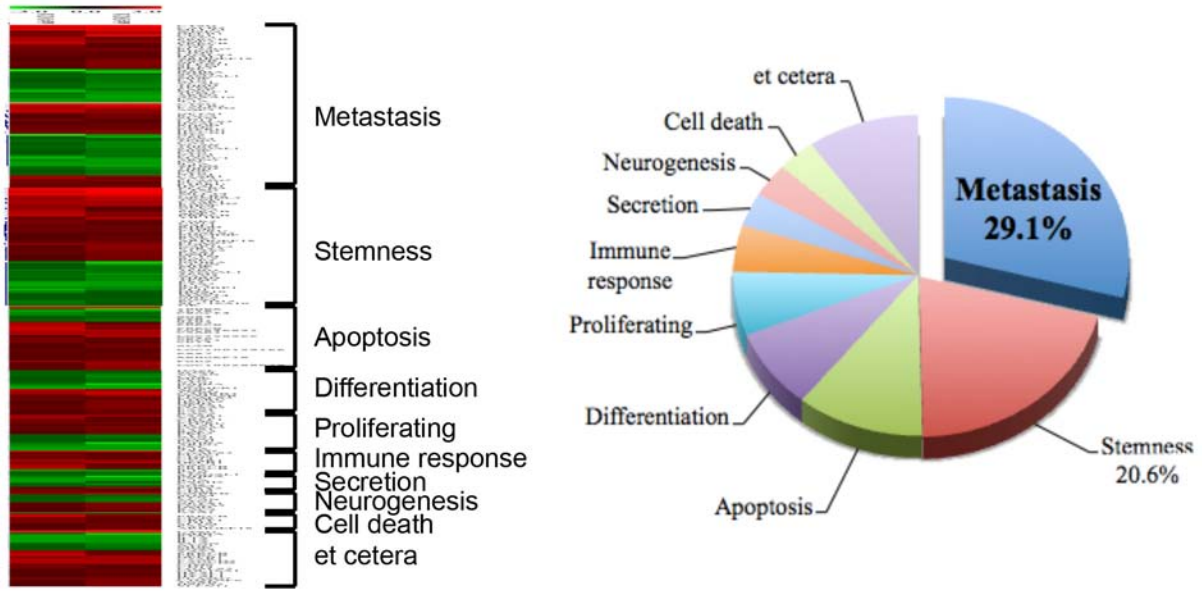

Supplementary Figure S2: Heat-map of significantly altered gene in shGal#1 and shGal#3 was categorized (left panel). Percentage of altered gene in each category was graphically presented as a pie graph (right panel).

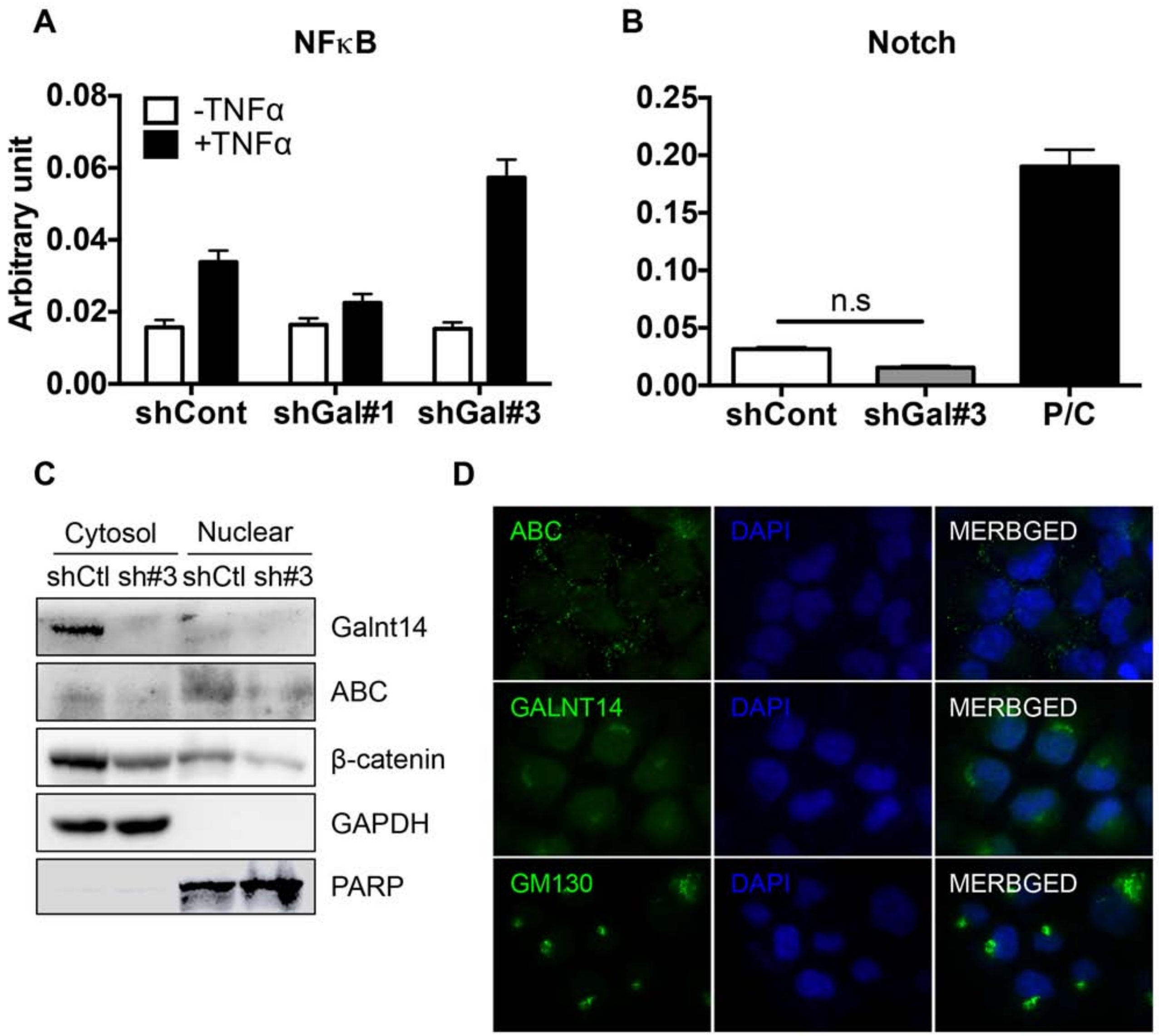

Supplementary Figure S3: Reporter activity of NF-kB with or without TNF-α treatment A. and Notch **B.** was determined by luciferase assay (n.s: not significant). **C.** Nuclear and cytoplasmic ABC and β-catenin level were determined by immunoblotting analysis. Immunoblotting for PARP served as a nuclear loading control and GAPDH was used as a cytoplasmic loading control. **D.** H460 cells were stained with ABC antibody, GalNAc-T14 antibody and GM130 antibody respectively (green). DAPI (blue) was used for nuclear counterstaining.

A

Target ID	Symbol	Definition	Accession	Fold_shGal-H460/WT shGal#1/ WT	shGal#3/ WT
A_23_P110531	FST	Follistatin	NM_013409	0.2910	0.2802
A_23_P144959	VCAN	versican - chondroitin sulfate proteoglycan 2 : CSPG2	NM_004385	0.4479	0.3530
A_23_P259071	AREG	Amphiregulin (schwannoma-derived growth factor)	NM_001657	0.3448	0.2712
A_23_P27013	HOXB9	Homeo box B9	NM_024017	0.5335	0.4145

B

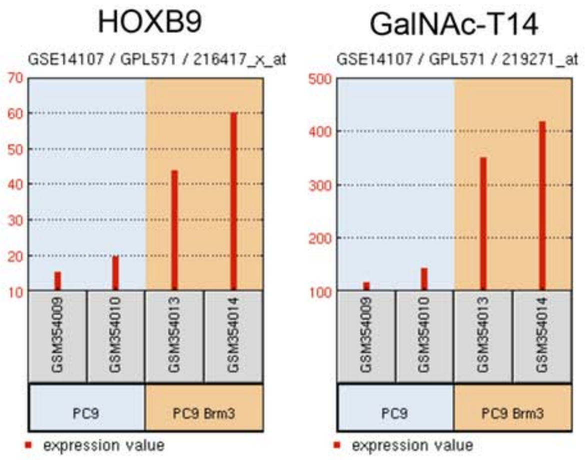

Supplementary Figure S4: A. List of genes of putative Wnt downstream target, altered in shGal#1 and shGal#3 was shown in a table. **B.** Relative expression level of HOXB9 and GalNA-T14 expression from GEO analysis of GSE14107 (between PC9 and PCR Brm3 cell line) was shown.

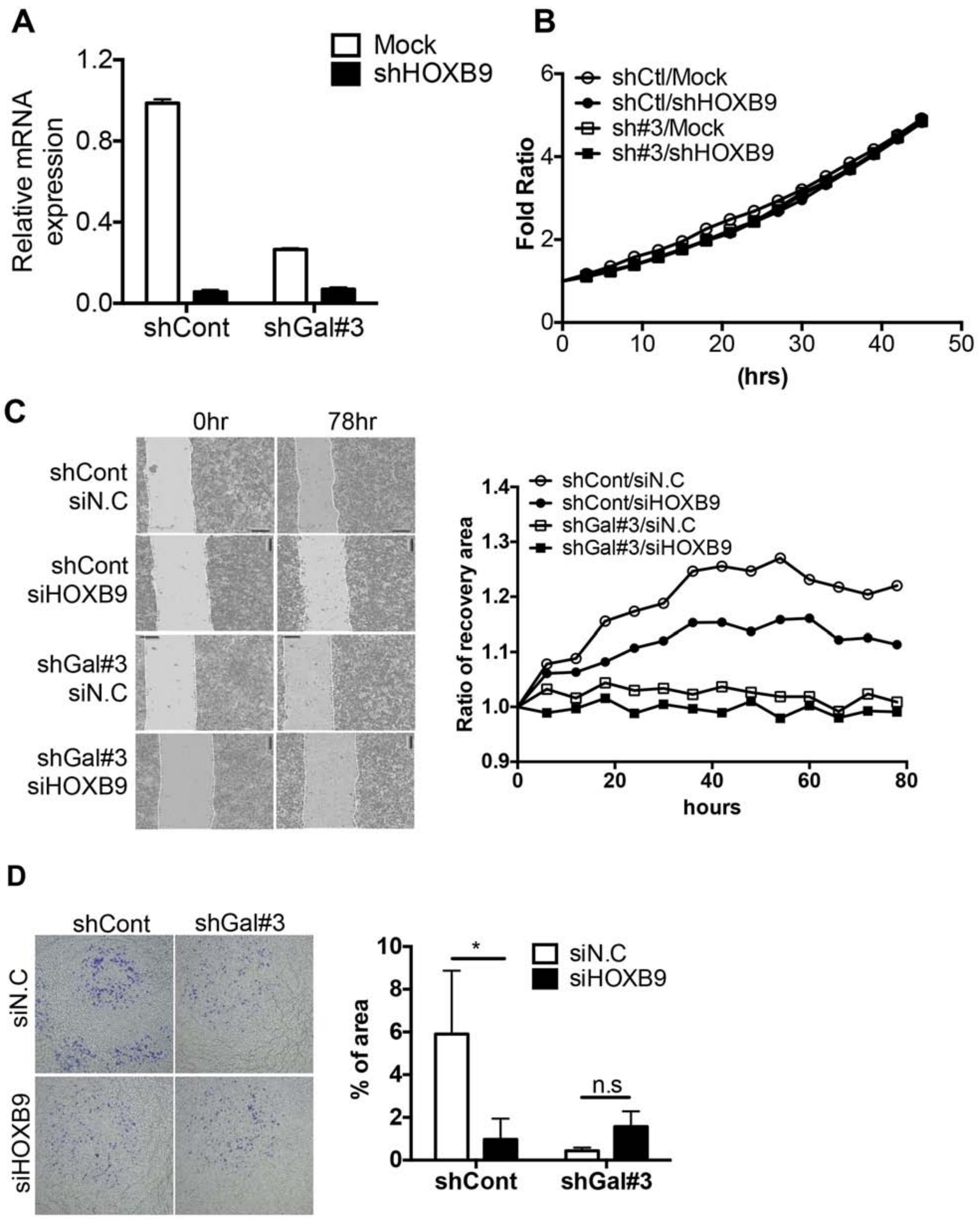

Supplementary Figure S5: A. mRNA level of HOXB9 was determined by real-time PCR analysis. **B.** Time dependent cell proliferation rate in each indicative cell line was shown. **C.** Representative images of recovery were shown after 78 hrs (left panel). Time dependent migration rate presented as a graph (right panel). **D.** Representative image of cells, invaded through trans-well membrane (left panel) was shown. Quantification of invaded cells was shown in a bar graph (right panel) (n.s: not significant, *n* = 5).

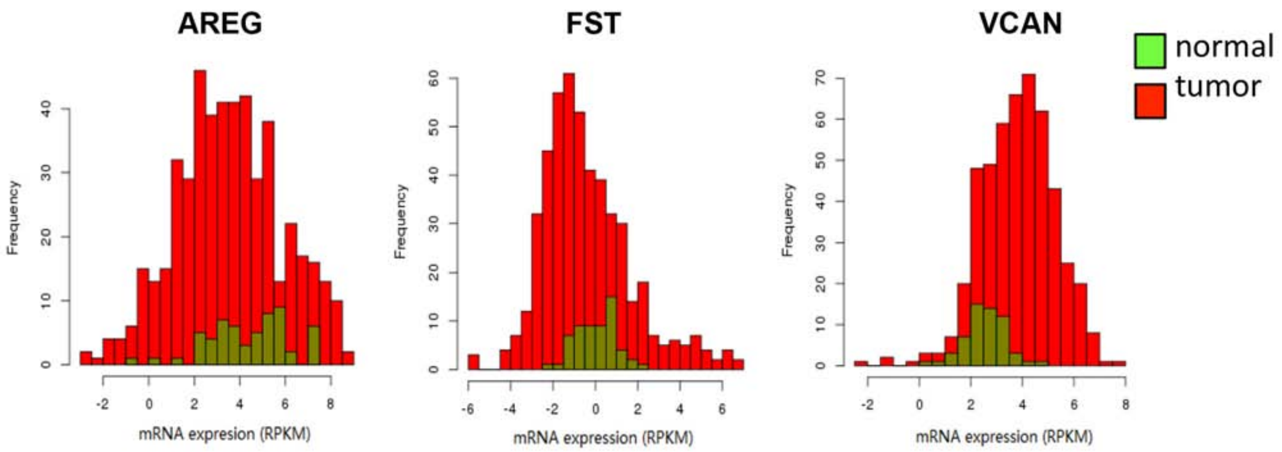

Supplementary Figure S6: Histograms of expression levels of candidate genes of AREG, FST, and VCAN from TCGA database analysis was shown. Green or red box indicates normal or tumor respectively.

Supplementary Table S1: Multivariate Cox regression analysis for overall survival (A or C) and relapse-free survival (B or D) of GalNAc-T14 (A and B) and HOXB9 (C and D) from TCGA database analysis

A

	n	**coef**	**se (coef)**	**95% CI**	**Hazard Ratio**	***p* value**
gender						
F	250				1.00	
M	210	0.041	0.173	0.742–1.463	1.042	0.814
age						
<65	218				1.00	
≧65	242	0.349	0.177	1.003–2.003	1.417	0.048
stage						
I	250				1.000	
II	110	0.721	0.221	1.335–3.170	2.057	0.001
III+IV	100	1.237	0.204	2.309–5.137	3.444	<0.001
**GALNT14**						
**low**	**195**				**1.000**	
**high**	**265**	**0.549**	**0.184**	**1.208–2.480**	**1.731**	**0.003**

B

	n	**coef**	**se (coef)**	**95% CI**	**Hazard Ratio**	***P***
gender						
F	165				1.00	
M	135	0.118	0.184	0.785–1.614	1.119	0.520
age						
<65	144					
≧65	156	0.203	0.185	0.853–1.759		0.272
Stage						
I	170				1.000	
II	73	0.639	0.212	1.250–2.870	1.864	0.003
III+IV	57	0.664	0.234	1.227–3.074	1.910	0.005
**GALNT14**						
**low**	**121**				**1.000**	
**high**	**179**	**0.407**	**0.191**	**1.035–2.183**	**1.487**	**0.033**

C

	n	**coef**	**se (coef)**	**95% CI**	**Hazard Ratio**	***p***
gender						
F	250				1.000	
M	210	0.023	0.173	0.728–1.437	1.023	0.896

	n	coef	se (coef)	95% CI	Hazard Ratio	*p*
age						
< 65	242				1.000	
≧65	218	0.342	0.177	0.995–1.991	1.407	0.054
stage						
I	250				1.000	
II	110	0.618	0.225	1.193–2.883	1.855	0.006
III+IV	100	1.234	0.203	2.306–5.120	3.436	<0.001
**HOXB9**						
**low**	**253**				**1.000**	
**high**	**207**	**0.426**	**0.177**	**1.084–2.165**	**1.532**	**0.016**

D

	n	coef	se (coef)	95% CI	Hazard Ratio	*p*
gender						
F	165				1.000	
M	135	0.097	0.184	0.769–1.580	1.102	0.596
age						
<65	144				1.000	
≧65	156	0.188	0.185	0.839–1.735	1.207	0.311
stage						
I	170				1.000	
II	73	0.538	0.220	1.114–2.634	1.713	0.014
III+IV	57	0.640	0.235	1.197–3.005	1.897	0.006
**HOXB9**						
**low**	**168**				**1.000**	
**high**	**132**	**0.323**	**0.190**	**0.952–2.006**	**1.381**	**0.089**
